# Cell:cell adhesion in sweet cherry fruit primarily due to pectins

**DOI:** 10.3389/fpls.2025.1690728

**Published:** 2026-01-30

**Authors:** Elsa Culemann, Christine Schumann, Andreas Winkler, Simon Sitzenstock, Moritz Knoche

**Affiliations:** Institute for Horticultural Production Systems, Leibniz-University Hannover, Hannover, Germany

**Keywords:** calcium, cellulase, cellulose, hemicellulase, hemicellulose, homogalacturonan, pectinase, *Prunus avium*

## Abstract

Rain cracking limits the production of sweet cherries wherever they are grown. Cracking occurs by the separation of neighboring cells along their cell walls (by cell:cell separation). The objectives were to identify the cell wall components involved in cell:cell adhesion using immunolabeling and monoclonal antibodies (mAbs) and digestion assays employing pectinases, hemicellulases, and a cellulase. The mAbs identified homogalacturonans (LM19 and LM20), arabinans (LM6), and (to a lesser extent) xyloglucans (LM25) in skin, parenchyma, xylem, and phloem. Galactan (LM5) occurred only in the phloem, and xylan/arabinoxylan (LM11) occurred only in the xylem. Digestion of parenchyma by polygalacturonase (PGase) increased with time and concentration. Throughout development, digestion was highest by galactanase (GALase), followed by PGase and pectate lyase (PLase). Digestion by the hemicellulases xylanase (XYLase), xyloglucanase (XGase), and mannanase (MANase) was lower. The lowest digestion was due to cellulase (CELase). Incubation in pectinases released more cells, protoplasts, and cell wall fragments than incubation in hemicellulases or CELase. Storage duration had no effect on the digestion of cell walls. Digestion of parenchyma by native enzymes from fruit juice was similar to that by purified enzymes. Boiling juice to destroy enzyme decreased digestion. Adding Ca reduced digestion by PGase. Extracting and complexing Ca by EGTA increased digestion. Across eight cultivars, GALase, PGase, and PLase digested more cell walls than hemicellulases or cellulase. Within pectinases, GALase, PGase, and PLase were equally effective in “Adriana”, whereas in “Kordia”, PGase was more effective than GALase and PLase. In “Flamengo Srim”, “Regina”, and “Staccato”, digestion by GALase, PGase, and PLase was low. We conclude that pectins are primarily responsible for cell:cell adhesion in sweet cherry fruit. Further studies should therefore explore the relationship between cell wall chemistry, Ca status, and cell:cell adhesion.

## Introduction

Rain cracking of sweet cherry fruit is a common problem in all areas of the world where the fruit is exposed to rain or high humidity during maturation and ripening. Exposure to rain reduces harvest quality and quantity of sweet cherry fruit (*Prunus avium* L.), because of macroscopic and microscopic cracking. Macroscopically cracked fruit is easily recognized by the naked eye due to deep cracks that extend deep into the flesh, sometimes to the pit. Fruit with macroscopic cracks is typically left in the orchard and never reaches the market chain. However, macroscopic cracking is always preceded by microscopic cracking, which is difficult to recognize without a microscope. Microcracks are minute fractures in the cuticle that impair its barrier functions, resulting in greatly increased water movement, into and out of the fruit, and an increased incidence of fruit rots (Christensen, 1996; Knoche and Winkler, 2017). Microcracking is the first step in a complex series of events that culminate in macrocracking. Based on the “Zipper” model (Brüggenwirth and Knoche, 2017; Schumann et al., 2019; Winkler et al., 2016), microcracks result from the cessation of cuticle deposition at the end of stage I/beginning of stage II development when the pit begins to harden and the fruit fresh mass is approximately 1 to 2 g (Alkio et al., 2012; Knoche et al., 2004). The subsequent stage III development is characterized by rapid increases in both fruit mass and surface area (Lilleland and Newsome, 1934; Tukey, 1934). This increase in fruit surface area now must occur in the absence of deposition of any new cuticular material, meaning a constant mass of cuticle must now be distributed over an increasing area of fruit surface (Knoche et al., 2004; Lai et al., 2016). Hence, the cuticle becomes thinner and more strained. Strain fractures soon appear (Grimm et al., 2012; Peschel and Knoche, 2005). At this stage, exposure of the fruit surface to moisture or high humidity exacerbates microcracking (Knoche and Peschel, 2006). A microcrack effectively bypasses the cuticle as a penetration barrier. Water flows more readily into the fruit, causing the cells of the outer flesh to burst (Grimm et al., 2019). These cells have a more negative osmotic potential than those of epidermis and hypodermis (Grimm and Knoche, 2015). The bursting of cells releases malic acid into the apoplast (Grimm et al., 2019). The malic acid extracts Ca from cell walls, weakening them and increasing the permeability of the membranes of the adjacent cells, causing further protoplasmic leakage (Winkler et al., 2015). The loss of turgor associated with these events permits cell wall swelling, which weakens the cell:cell adhesion (Brüggenwirth and Knoche, 2017; Schumann et al., 2020). The cells separate schyzogenously, i.e., along their middle lamellae (Brüggenwirth and Knoche, 2017; Schumann et al., 2019). Stress concentration at the crack tip and the tension in the surrounding skin causes a crack to propagate (“run”) and the microcrack deepens and extends to form a macrocrack (Grimm et al., 2012; Schumann et al., 2019). It is stage III development, where the susceptibility of sweet cherry fruit to cracking increases (Christensen, 1973).

Surprisingly little information is available on the chemical composition of the cell walls in sweet cherry fruit (Basanta et al., 2013, Basanta et al., 2014; Fils-Lycaon and Buret, 1990; Lahaye et al., 2021; Roy et al., 1992; Salato et al., 2013; Schumann et al., 2020). Most of this information is based on isolated cell walls *in vitro* involving selective extraction of tissue using solvents (Schumann et al., 2020; Sozzi et al., 2002). Unfortunately, this procedure destroys the native *in vivo* structure of the cell wall and, hence, does not permit spatial resolution. Robust conclusions concerning the cell wall fractions responsible for cell:cell adhesion cannot reasonably be drawn. Schumann et al. (2019) used immunolabeling to identify demethylated homogalacturonan (HG) on the surfaces of cells exposed by microcracking in sweet cherry. The stained homogalacturonan clearly outlined the cells that separated along their cell walls (rather than through cell walls). Additionally, Roy et al. (1992) reported demethylated HG in the middle lamella during stage II and in the entire cell wall during stage III. This observation suggests failure of the HG of the middle lamellae as the primary mode of failure in cracking. Confirmation of this hypothesis may be gained by investigating cell:cell adhesion using highly specific, purified cell wall-degrading enzymes to digest sweet cherry tissue. The extent of degradation and the nature of the fragments separated from the tissue are likely to permit conclusions about the cell wall fractions involved in cell:cell adhesion [see Ordaz-Ortiz et al. (2009) for tomato].

The objectives of our study were (1) to identify and localize the major cell wall fractions of sweet cherry fruit using immunolabeling and monoclonal antibodies (mAbs) and (2) to identify the cell wall components responsible for cell:cell adhesion using digestion assays and highly specific pectinases, hemicellulases, and a cellulase.

## Materials and methods

### Plant material

Sweet cherries (*P. avium* L.) of the cultivars Adriana, Burlat, Dönissens Gelbe, Flamengo Srim, Kordia, Regina, Sam, Staccato, and Sweetheart were harvested in 2021 and 2022 from trees growing in a greenhouse or in the field under a rain shelter at the Horticultural Research Station of the Leibniz University in Ruthe (latitude 52°14′ N, longitude 9°49′ E) and the Herrenhausen Campus of Leibniz University (latitude 52°27′ N, longitude 09°84′ E). All trees were grafted on Gisela 5 rootstocks except for “Sweetheart”, which were grafted on Gisela 3 rootstocks. All experiments were carried out using fully mature fruit (end of stage III, Lilleland and Newsome, 1934; Tukey, 1934). At this stage, the fruit is at commercial maturity based on color, size, and taste. The only exception was the developmental time course in “Sam” fruit where samples were taken between 44 (end of stage II) and 87 days after full bloom (DAFB) (overmature stage III). Fruit were selected for uniformity of size and color. Most fruit was processed immediately after harvest. When this was not possible, fruit were held in cold storage at 2°C for no longer than 3 days. Mass, color, and osmolarity were determined. The color was measured using a colorimeter (CM-2500d; Konica Minolta, Marunouchi, Japan). The osmolarity of juice extracted from 10 cherries was measured using vapor pressure osmometry (Vapro 5520 and 5600; Wescor, Logan, UT, USA).

### Immunofluorescence microscopy

Mature fruits of the cultivars Adriana (greenhouse) and Sam (rain shelter) were fixed in Karnovsky fixative solution (Karnovsky, 1965) and stored at 12 °C in a cold room. For paraffin embedding, five tissue blocks (3 × 3 × 3 mm) were cut from five fruit using a razor blade. The blocks contained cuticle, epidermis, hypodermis, parenchyma, and vascular bundles.

Blocks were washed in deionized water followed by 65% (v/v) aqueous ethanol for 48 h to remove the fixative solution. The blocks were then dehydrated in an ascending series of aqueous ethanol [70%, 70%, 80%, 90%, and 96% (v/v) for 10, 10, 15, 30, and 30 min each] followed by 100% isopropanol for 40 min twice. For infiltration, the blocks were incubated twice for 40 min each in xylene substitute (ROTICLEAR^®^; Roth, Karlsruhe, Germany), followed by a 1:1 mixture of xylene substitute and paraffin (ROTI^®^Plast; Roth) for 40 min, and paraffin only (ROTI^®^Plast; Roth) for 40 min (all at 62°C). All dehydration and infiltration steps were carried out under a mild vacuum (10.8 kPa). The blocks were held at 4 °C until sectioning.

The blocks were sectioned (7 µm thickness) using a rotary microtome (Hyrax M 55; Zeiss, Oberkochen, Germany), and the sections were transferred to a microscope slide. The slides were dried at 40°C overnight. The paraffin was extracted using xylene substitute (2 × 10 min), and the sections were rehydrated using aqueous ethanol [98%, 80%, 70%, and 60% (v/v); for 10 min each] and deionized water (2 × 10 min) at ambient temperature. The sections were then dried in an oven overnight at 40°C.

For immunolabeling, sections were processed following the protocol of Avci et al. (2012) and the modifications of Ordaz-Ortiz et al. (2009) and Schumann et al. (2019). Non-specific binding sites were blocked using 3% (w/v) bovine serum albumin (BSA) prepared in phosphate-buffered saline (PBS) for 30 min. The blocking solution was removed and the section was washed 3 × 5 min in PBS. Primary monoclonal rat antibodies (mAbs; PlantProbes, Leeds, UK) were prepared in a fivefold dilution in PBS buffer at pH 7.0. The primary mAbs reacting with pectins were LM5 (anti-galactan) (Jones et al., 1997), LM6 (anti-arabinan) (Willats et al., 1998), LM7 (anti-non-blockwise methylated HG) (Willats et al., 2001), LM8 (anti-xylogalacturonan) (Willats et al., 2004), LM19 (anti-demethylated HG) (Verhertbruggen et al., 2009), and LM20 (anti-blockwise methylated HG) (Verhertbruggen et al., 2009). The primary mAbs reacting with hemicelluloses were LM11 (anti-xylan/arabinoxylan) (Mccartney et al., 2005), LM21 (anti-mannan) (Marcus et al., 2010), and LM25 (anti-xyloglucan) (Pedersen et al., 2012). Solutions containing the primary mAbs were removed after 60 min, and the sections were washed 3 × 5 min using PBS. The primary antibodies bound to the respective cell wall epitopes of the section were now labeled with a secondary antibody against rat immunoglobulin that carried the fluorescence marker (Alexa Fluor™ 488 anti-rat; Thermofisher, Darmstadt, Germany). The secondary antibody was diluted 100-fold in PBS containing 3% BSA, applied to the section on the microscope slide, and incubated for 1.5 h. All subsequent steps were carried out in the dark. Sections were washed 3 × 5 min in PBS and then stained with 0.05% (w/v) toluidine blue (Merck, Darmstadt, Germany) for bright-field microscopy and suppression of autofluorescence. Sections were then rinsed with deionized water. All incubation steps were conducted in a closed polyethylene box at 100% relative humidity (RH).

The stained sections were transferred to the stage of a fluorescence microscope [BX-60 with U-MWB filter (450–480 nm excitation, ≥ 520 nm emission); Olympus, Hamburg, Germany] and viewed at ×100. Images were captured with a camera (DP73; Olympus) and processed using image analysis (cellSens v1.7.1; Olympus). For imaging, exposure times for bright-field and fluorescence microscopy were held constant at 3 ms and 1 s, respectively.

### Digestion assays

Digestion assays were carried out following the protocol of Ordaz-Ortiz et al. (2009) with minor modifications. Tissue blocks were prepared using the fleshy parenchyma (mesocarp) of sweet cherries unless otherwise specified. The skin comprising epidermis, hypodermis, and some adhering flesh was removed using a razor blade and the parenchyma was cut into 1- to 2-mm blocks (cubes) using a custom multi-blade ribbon cutter. A sample of 2.5 g of blocks prepared from five fruit was transferred into pre-dried (70 °C for 24 h) and pre-weighed 50-mL centrifuge tubes (AN78.1; Roth, Karlsruhe, Germany) and suspended in 20 mL of isotonic digestion medium. The osmolarity of the digestion medium was adjusted using glucose. Unless otherwise specified, the digestion medium contained the following buffers at 7.5 U mL^−1^ of enzymes (Megazyme, Co. Wicklow, Ireland): endo-1,4-β-D-galactanase (GALase) (100 mM NaOAc + 1 mg mL^−1^ BSA, pH 4.0), endo-polygalacturonanase (PGase) (100 mM NaOAc + 1 mg mL^−1^ BSA, pH 5.5), pectate lyase (PLase) (50 mM CAPS in 1 mM CaCl_2_, pH 10.0), endo-1,4-β-xylanase M3 (XYLase) (100 mM phosphate + 1 mg mL^−1^ BSA, pH 6.0), xyloglucanase (XGase) (100 mM NaOAc + 1 mg mL^−1^ BSA, pH 5.5), β-mannanase (MANase) (100 mM glycine, pH 8.8), and endo-cellulase (CELase) (100 mM NaOAc + 0.5 mg mL^−1^ BSA, pH 4.5). Tissue blocks incubated in isotonic buffer at the respective pH but with no enzyme were used as controls. Samples were incubated for 23 h at 40°C on a shaker (Polymax 1040; Incubator 1000, Heidolph, Schwabach, Germany) set at 10 rpm. After 23 h, the content of the centrifuge tubes was sieved through a 450-µm mesh sieve (22363549; Fisher Scientific GmbH, Schwerte, Germany) and rinsed 4× with 20 mL of deionized water. Since some digestion also occurred in the buffer-only control, an additional control was used where freshly prepared tissue blocks were rinsed in 4 × 20 mL of deionized water—there was no incubation in buffer with or without enzyme for this additional control.

To determine the dry mass of the digested tissue, the sieved samples were dried at 70 °C for 7 days and weighed (CPA225D; Sartorius, Göttingen, Germany). The percentage of digestion was calculated as follows. First, the dry matter content (DMC; %) was calculated using (Equation 1) where DM and FM refer to the dry mass (g) and fresh mass (g), respectively.

(1) $$DMC\;=\frac{DM}{FM}*100$$

The gross digestion in the isotonic buffered enzyme solution (*Digestion_buffer + enzyme_*) was calculated from (Equation 2), where *DMC_buffer + enzyme_* is the dry matter content of samples after incubation in isotonic buffer plus enzyme and *DMC_water_* is the dry matter content of samples rinsed in 4 × 20 mL of deionized water.

(2) $$Digestion_{\;buffer+\;enzyme}=\;100-\;\frac{DMC_{buffer+enzyme}\;}{DMC_{water}}*100$$

Some digestion occurred in the presence of the isotonic buffer only (no enzymes added). The percentage of digestion due to the buffer plus any endogeneous enzymes that are present in the tissue was calculated from (Equation 3) where *DMC_buffer_* represents the dry matter content of samples after incubation in isotonic buffer only (no enzymes) and *DMC_water_* is the dry matter content of samples rinsed 4 × in 20 mL of deionized water.

(3) $$Digestion_{buffer}=\;100-\;\frac{DMC_{buffer}}{DMC_{water}}*100$$

The digestion due to the added enzymes (*Digestion_Enzyme_*) was calculated from (Equation 4):

(4) $$Digestion_{enzyme}=\;Digestion_{buffer\;+\;enzyme}-\;Digestion_{buffer}$$

This procedure assumes a simple additive effect of added enzymes and does not account for potential interactions between endogenous and added enzymes. However, we are not aware of any reports of such interactions in sweet cherry, and therefore, we assumed no interaction as a first approximation. All digestion assays were carried out using three replicates.

### Experiments

The time course of enzyme digestion was determined using PGase (50 mM NaOAc, pH 4.5) and mature greenhouse-grown “Adriana”. Samples were incubated for 0, 4, 10, 22, 46, or 72 h.

The concentration response was established for PGase (50 mM NaOAc, pH 4.5) using mature greenhouse-grown “Adriana”. Enzyme concentrations were 0, 0.5, 1.5, 5, or 15 U mL^−1^ PGase.

The developmental time course was determined using greenhouse-grown “Sam”. Fruit were harvested 44, 50, 57, 64, 72, 79, or 87 DAFB. Digestion assays were conducted using the pectinases GALase, PGase, and PLase; the hemicellulases XYLase, XGase, and MANase; and the cellulase CELase.

Potential changes in cell walls during storage were investigated using fruit of greenhouse-grown mature “Staccato”. Fruit were held in cold storage at 2 °C and 100% RH for 1, 8, 15, or 33 days. To prevent dehydration and to simulate packaging, the cherries were packed in a polyethylene bag that remained unsealed to prevent anaerobiosis. Enzyme digestion was performed at a concentration of 7.5 U mL^−1^ as described above using the following buffers and enzymes: GALase (50 mM NaOAc, pH 4.5), PGase (50 mM NaOAc, pH 4.5), PLase (50 mM CAPS in 2 mM CaCl_2_, pH 10.0), XYLase (50 mM phosphate, pH 6.5), XGase (50 mM NaOAc, pH 4.5), MANase (100 mM glycine, pH 8.8), and CELase (50 mM NaOAc, pH 4.5). The buffer systems used in this experiment differed from the one specified above because the enzymes originally used were discontinued and the recommendations of the manufacturer had changed with the new products.

The effect of cultivar was investigated by comparing digestion of tissue blocks prepared from mature greenhouse-grown “Adriana”, “Burlat”, “Dönissens Gelbe”, “Flamengo Srim”, “Kordia”, “Regina”, “Sam”, and “Staccato”. Enzymes and buffers were used as described under the “Digestion assays” section. Fruit mass, osmolarity of the juice, dry matter content, and color are summarized in **Supplementary Table S1**.

To quantify digestion of sweet cherries by enzymes naturally present in the fruit, the digestion of tissue blocks from mature “Sweetheart” was measured using juice extracted from the same batch of “Sweetheart” fruit. Tissue blocks were incubated in 20 mL of juice, 20 mL of juice that had been boiled for 5 min to destroy any enzymes present in the juice, or 20 mL of a synthetic juice prepared using the five major osmolytes of sweet cherry (Herrmann, 2001). For example, for a synthetic juice having an osmotic potential of −2.5 MPa, molar concentrations were as follows: 68 mM malic acid, 420 mM glucose, 383 mM fructose, 75 mM sorbitol, and 55 mM KOH. The juice was centrifuged (3 × 7,000 *g* for 7.5 min) and the supernatant was taken for incubation. KOH was used to adjust the pH value.

The effect of calcium (Ca) on digestion of mature field-grown “Regina” by PGase was studied by adding 10 mM CaCl_2_ to the buffered PGase solution or by extracting Ca from cell walls by adding 5 mM ethylene glycol-bis(β-aminoethyl ether)-N,N,N′,N′-tetraacetic acid (EGTA) to the buffered PGase solution. The digestion assay was carried out as described above.

The effect of pre-incubating mature “Sweetheart” fruit in CaCl_2_ solution was also investigated. Fruit were harvested from the greenhouse, and the pedicel cut and the pedicel cavity including the pedicel end were sealed with silicone rubber (Dowsil™ SE 9186 Sealant; Dow, Midland, USA). Thereafter, fruit were incubated for 18 h in an isotonic solution containing 100 mM CaCl_2_. Glucose was used as an osmoticum to adjust the osmolarity of CaCl_2_ solution. Fruit incubated in the same solution without CaCl_2_ served as control. Fruit that cracked during the pre-incubation were discarded. Following pre-incubation, tissue blocks were prepared and digestion was quantified in PGase solution buffered with 50 mM NaOAc at pH 4.5 as described above.

Cell fragments obtained in digestion assays of greenhouse-grown “Regina” sweet cherry were inspected by light microscopy. Tissue blocks were prepared and digestion assays were carried out as described above. Ten milliliters of the digestion medium was sieved (40 µm mesh width), and the cell fragments on the sieve were transferred into a 2-mL reaction vessel containing 2 mL of the respective isotonic buffer and 0.05% (w/v) toluidine blue. The reaction vials were gently shaken. A 10-µL aliquot of the solution was transferred onto a microscope slide and viewed at ×100 by light microscopy (BX-60; Olympus). Three random images of the solution (wetted area on the microscope slide approximately 24–35 mm^2^) were taken (DP73; Olympus), and the area occupied by the cell wall fragments was quantified using image analysis (cellSens 1.7.1; Olympus). The number of intact cells or protoplasts was counted. Subsequently, the tissue blocks were removed from solution and photographed under a binocular microscope (MZ10F; Leica Microsystems, Wetzlar, Germany) (DP73; Olympus).

### Data analyses

Data in tables and figures are presented as means ± standard errors (SEs). Mean comparison and linear regression analyses were carried out using R (version 4.2.2; R Foundation for Statistical Computing, Vienna, Austria). Continuous data were analyzed by analysis of variance using a linear model (lm). Percentage digestion data were arcsine transformed to obtain normal distributions before analysis of variance. For discrete data, a generalized model with a Poisson distribution was used. Means were compared using the Tukey test (package emmeans 1.8.2 with function emmeans, https://CRAN.R-project.org/package=emmeans). The statistics software package SAS (version 9.1.3; SAS Institute, NC, USA) was used for correlation analysis.

## Results

### Immunofluorescence microscopy

**Figure 1:**
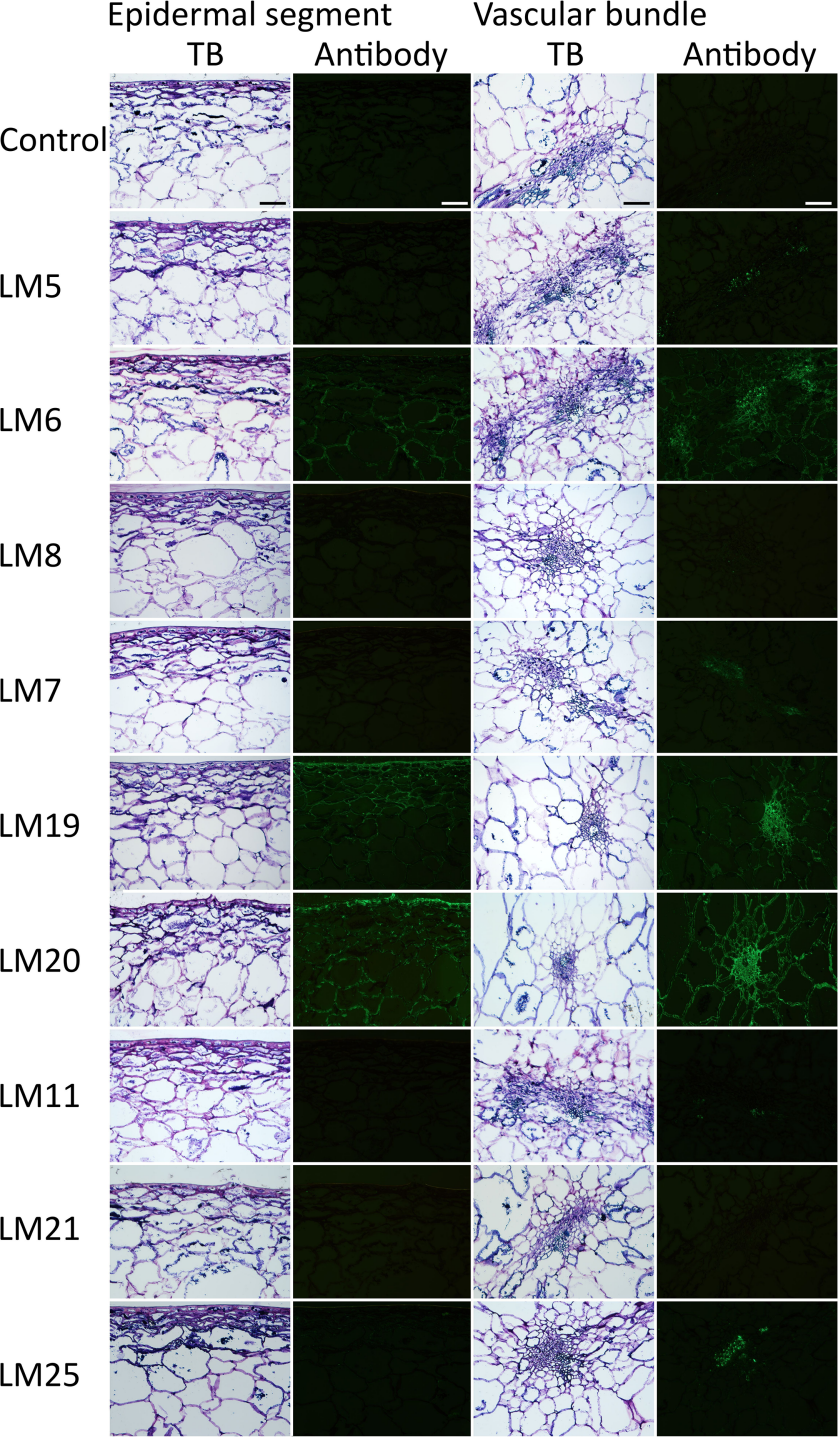
Immunolocalization of cell wall epitopes in the epidermis, vascular bundle, and parenchyma of mature “Sam” sweet cherry. Monoclonal antibodies (mAbs) were for the pectin fractions galactan (LM5), arabinan (LM6), xylogalacturonan (LM8), non-blockwise methylated homogalacturonan (HG) (LM7), demethylated HG (LM19), and blockwise methylated HG (LM20), and for the hemicellulose fractions xylan/arabinoxylan (LM11), mannan (LM21), and xyloglucan (LM25). Light micrographs taken in brightfield stained with toluidine blue (TB). Scale bar = 100 µm.

The antibodies bound to epitopes of the cell walls of “Adriana” and “Sam” sweet cherry (**Figures 1**; **Supplementary Figure S1**; **Table 1**).

**Table 1:** Intensity of binding of monoclonal antibodies (mAbs) against epitopes of pectins and hemicelluloses in cell walls of the sweet cherry cultivars “Adriana” and “Sam”.

Component	Fraction	Antibody	Adriana					Sam				
			ED	HD	Par	Xy	Ph	ED	HD	Par	Xy	Ph
Pectins	Galactan	LM5	−	−	−	−	++	−	−	−	−	++
	Arabinan	LM6	+	+	+	++	++	+	+	++	+	++
	Xylogalacturonan	LM8	−	−	−	−	−	−	−	−	−	+
	Non-blockwise methylated HG	LM7	−	−	+	+	++	−	−	−	+	++
	Demethylated HG	LM19	++	++	++	++	++	++	++	++	++	++
	Blockwise methylated HG	LM20	++	++	++	+	++	++	++	++	+	++
Hemicelluloses	Xylan/Arabinoxylan	LM11	−	−	−	++	−	−	−	−	++	−
	Mannan	LM21	−	−	−	−	−	−	−	−	−	−
	Xyloglucan	LM25	+	+	+	++	++	+	+	+	+	++

The intensity of binding of the mAbs was assessed for thin sections comprising epidermis (ED), hypodermis (HD), parenchyma (Par), and xylem (Xy) and phloem (Ph). The intensity of binding as indexed by the intensity of fluorescence was rated using a three-score rating scheme: no binding (−), weak binding (+), and strong binding (++). Fruit were at the fully mature stage. *N* = 5.

There was little difference between the two cultivars. The mAbs identified the pectins homogalacturonans (LM19 and LM20) and arabinans (LM6) in the skin, parenchyma, xylem, and phloem and (to a lesser extent) the hemicellulose xyloglucan (LM25). For arabinan and xyloglucan, binding was stronger to the vascular tissue than to the parenchyma or skin. Galactan (LM5) was present only in the phloem, and xylan and arabinoxylan (LM11) were present only in the xylem. There was no binding to mannan (LM21).

### Digestion assays

**Figure 2:**
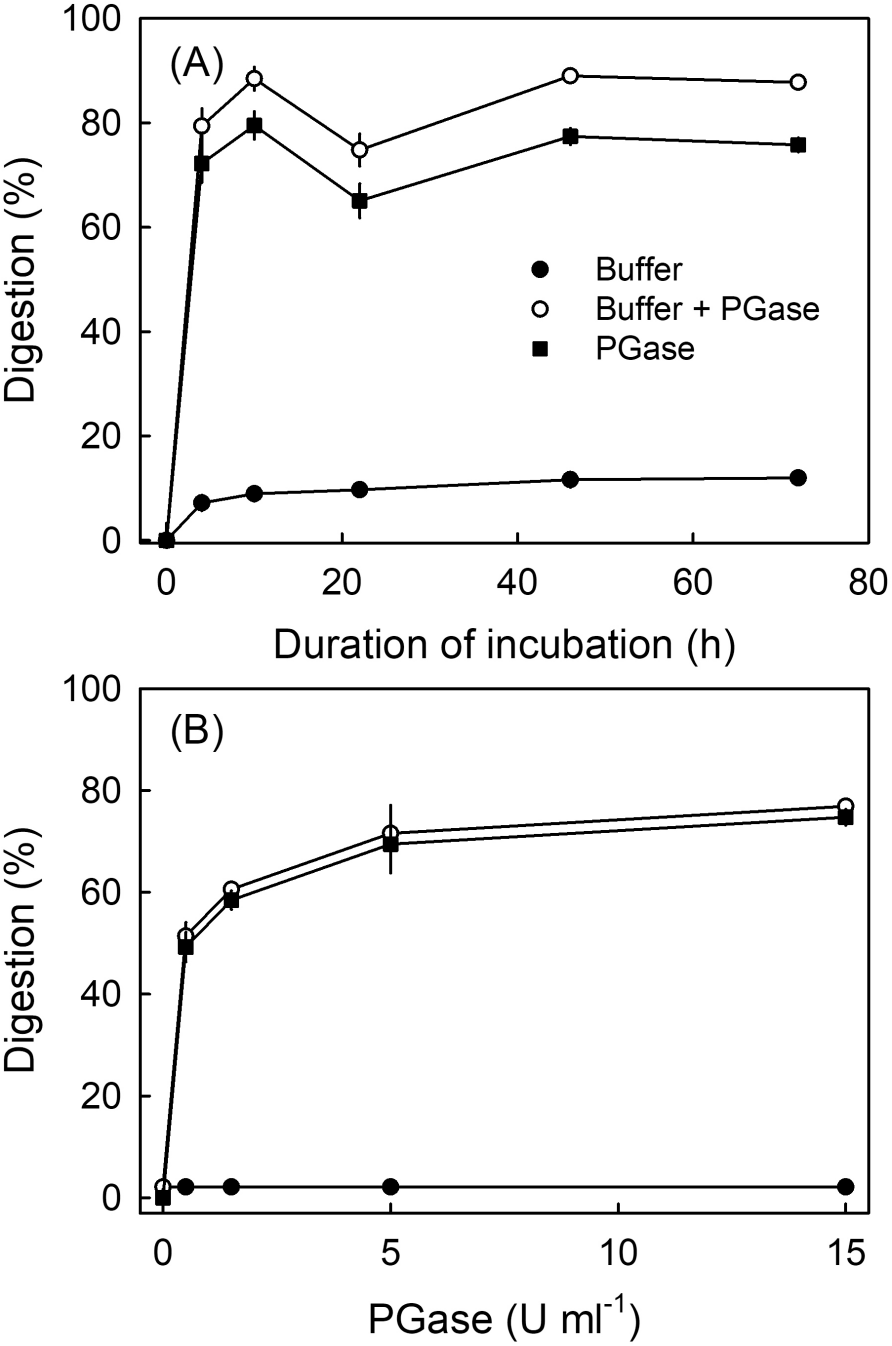
**(A)** Time course of digestion of parenchyma of mature “Adriana” sweet cherry incubated in endo-polygalacturonase (PGase). **(B)** Effect of concentration of PGase on digestion of parenchyma of mature “Adriana” sweet cherry. The enzyme solution was buffered using the manufacturer-recommended buffer system and pH. Buffer only (no enzyme) served as control. *N* = 3.

*Time course.* Digestion of parenchyma by PGase increased rapidly up to 4 h and then remained constant (**Figure 2A**).

*Concentration response.* Increasing the concentration of PGase increased digestion up to 5 U mL^−1^ PGase (**Figure 2B**). Higher PGase concentrations were not more effective. There was little digestion from buffer only as compared to buffer + PGase.

**Figure 3:**
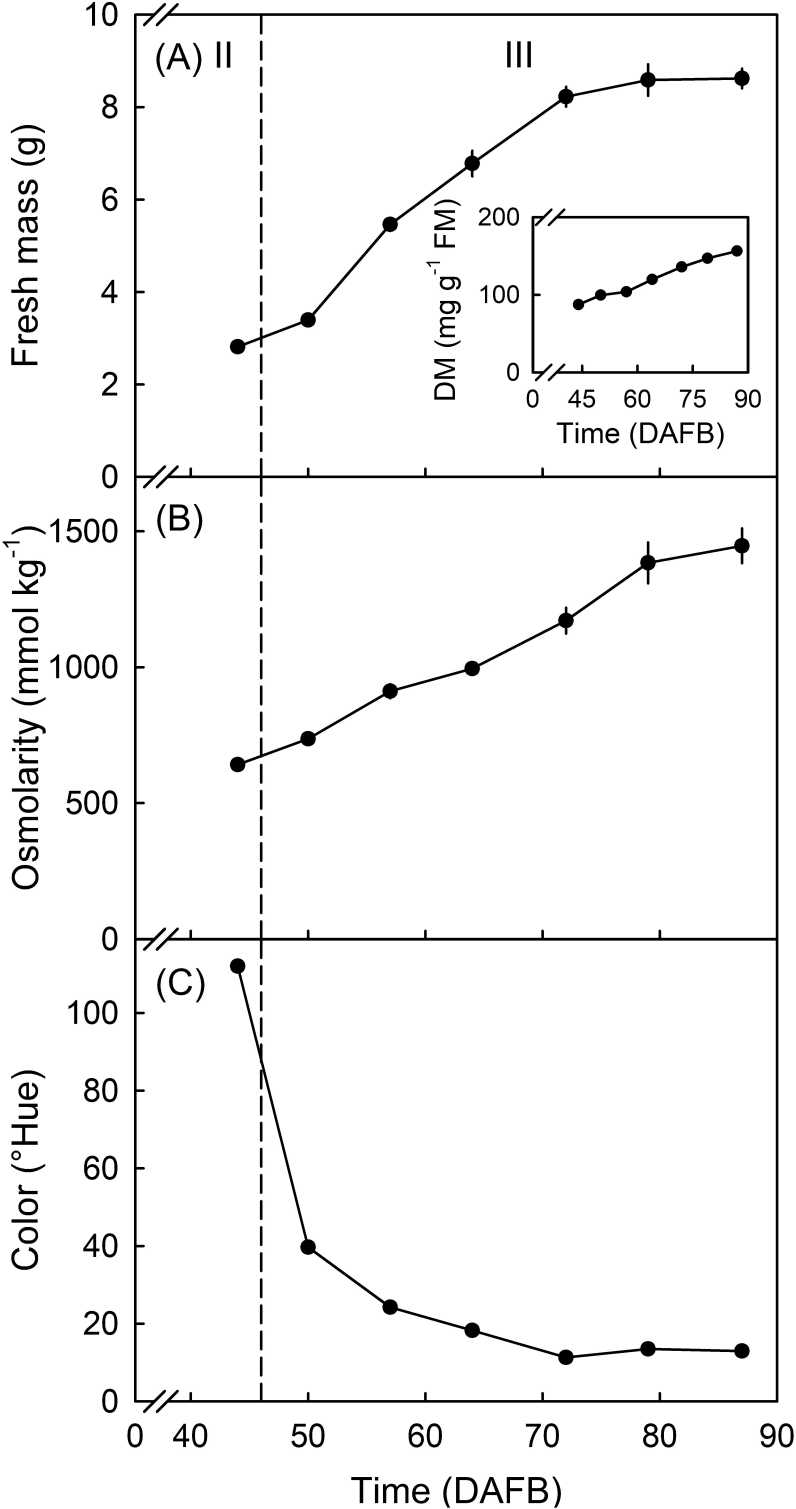
Developmental time course of change in fruit mass **(A)**, dry matter content (DM) **(A**, inset**)**, osmolarity **(B)**, and color as indexed by the hue angle **(C)** of “Sam” sweet cherry. *X* axis scale in days after full bloom (DAFB). The dashed line indicates the stage II/stage III transition (Lilleland and Newsome, 1934; Tukey, 1934). *N* = 10.

*Developmental time course.* The developmental time course revealed a rapid increase in fruit mass, dry matter content, and osmolarity between 50 and 78 DAFB. This resembled final swell during stage III development (**Figures 3A, B**).

Color change as indexed by a decrease in Hue angle began at 44 DAFB and continued until approximately 72 DAFB (**Figure 3C**). The decrease in Hue corresponded to a change from green to dark red. Based on these changes, the transition from stage II to stage III development occurred at approximately 46 DAFB.

**Figure 4:**
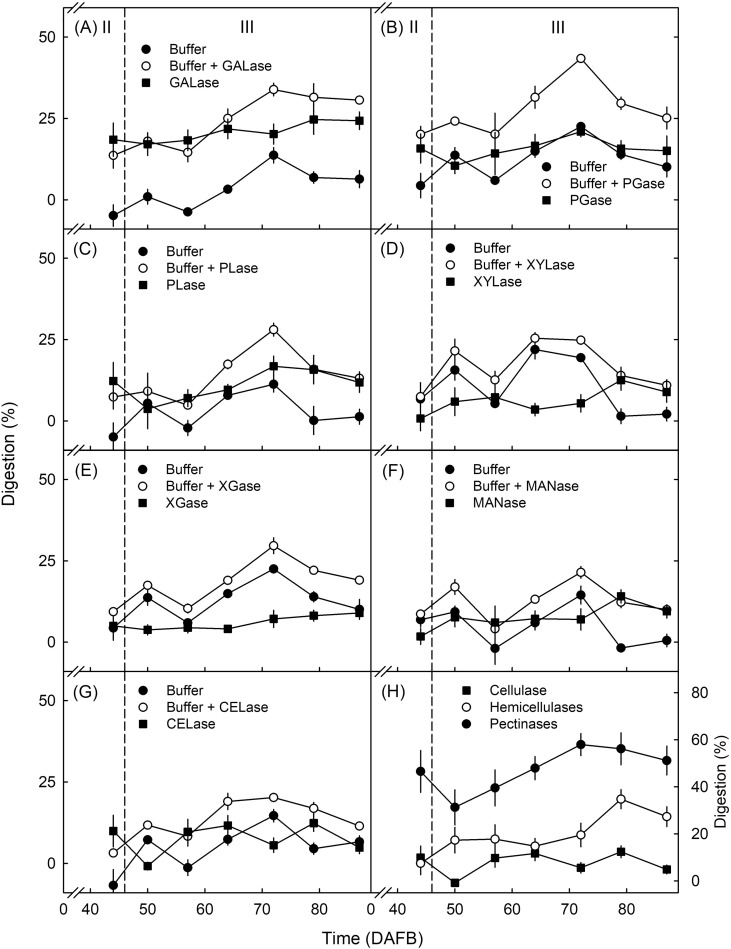
Effect of cell wall-degrading enzymes on the digestion of parenchyma of developing “Sam” sweet cherry. Enzymes for the pectin fractions were **(A)** endo-1,4-β-D-galactanase (GALase), **(B)** endo-polygalacturonase (PGase), and **(C)** pectate lyase (PLase). Enzymes for the hemicellulose fractions were **(D)** endo-1,4-β-xylanase M3 (XYLase), **(E)** xyloglucanase (XGase), and **(F)** β-mannanase (MANase). For the cellulose fraction, **(G)** endo-cellulase (CELase) was used. **(H)** Cumulative digestion by pectinases, hemicellulases, and cellulase. The fruit were sampled between 44 and 87 days after full bloom (DAFB). All enzyme solutions were buffered at their respective pH optima using the manufacturer-recommended buffer system and pH. Buffer only (no enzyme) served as control. *N* = 3.

Digestion was highest by GALase, followed by PGase and PLase (**Figures 4A, B, C**). For GALase, there was a consistent increase in digestion during development. Digestion by XYLase, XGase, MANase, and CELase was markedly lower (**Figures 4D–G**). Except for MANase, there was no consistent trend during development (**Figures 4D–F**).

Across all enzymes, digestion was higher for buffer + enzyme than for buffer alone (**Figures 4A–H**). Calculating cumulative digestion revealed that digestion by pectinases exceeded that by hemicellulases. The lowest digestion was due to cellulase (**Figure 4H**).

**Figure 5:**
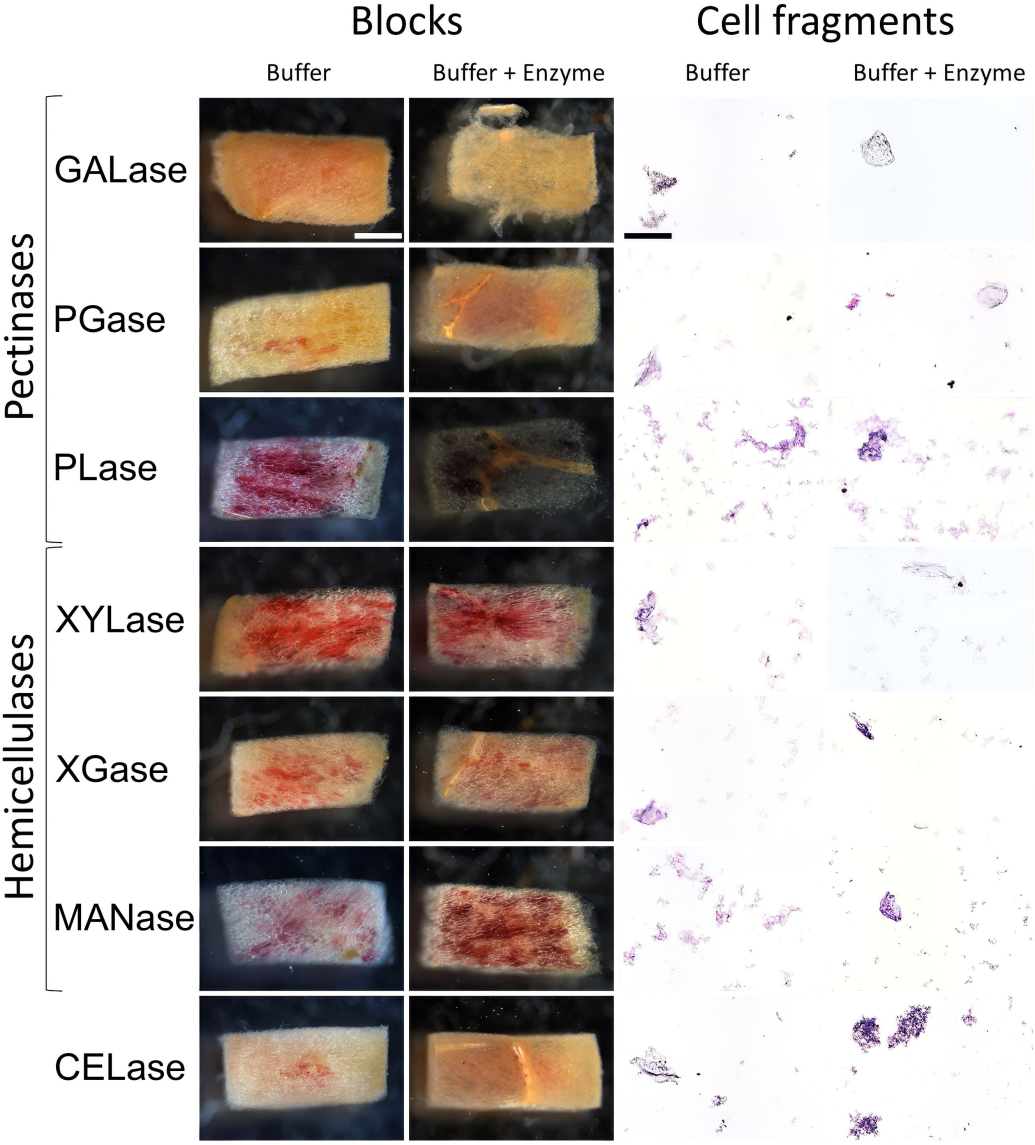
Photographs of digesting parenchyma blocks (left two columns) and cell fragments released during digestion (right two columns) in buffer (“buffer”) or buffer plus enzymes (“buffer + enzyme”) digestion solution of mature “Regina” sweet cherries. The pectin-degrading enzymes were endo-1,4-β-D-galactanase (GALase), endo-polygalacturonase (PGase), and pectate lyase (PLase). The hemicellulose-degrading enzymes were endo-1,4-β-xylanase M3 (XYLase), xyloglucanase (XGase), and β-mannanase (MANase). To degrade cellulose, an endo-cellulase (CELase) was used. All enzyme solutions were buffered using the manufacturer-recommended buffer system and pH. Buffer only (no enzyme) served as control. Cell wall fragments were stained with toluidine blue. Scale bar = 1 mm for blocks and 100 µm for cell wall fragments. *N* = 3.

*Microscopy of tissue blocks and cell fragments.* Incubating tissue blocks in the pectinases GALase, PGase, and PLase markedly altered the shape of the blocks. The cut edges lost shape during incubation. In contrast, tissue blocks incubated in hemicellulases or cellulase, or buffer only, maintained their shape with sharp edges (**Figure 5**).

Incubation in the pectinases also released more cells than in the hemicellulases or cellulase, producing the highest number of protoplasts and had the largest area fraction of released cell fragments (**Figure 5**; **Table 2**).

**Table 2:** Number of cells and protoplasts and cell wall fragments released from the parenchyma of mature “Regina” sweet cherry incubated in endo-1,4-β-D-galactanase (GALase), endo-polygalacturonase (PGase), pectate lyase (PLase), endo-1,4-β-xylanase M3 (XYLase), xyloglucanase (XGase), β-mannanase (MANase), or endo-cellulase (CELase).

Fraction	Treatment	Cells (*n*)			Protoplasts (*n*)			Fraction area (mm^2^)		
		Buffer + enzyme	Buffer	Enzyme	Buffer + enzyme	Buffer	Enzyme	Buffer + enzyme	Buffer	Enzyme
Pectinases	Total	10 ± 2	1 ± 1	9 ± 2	10 ± 3	3 ± 2	7 ± 4	1.6 ± 0.2	1.3 ± 0.3	0.3 ± 0.2
	GALase	3 ± 2 ab	1 ± 1 a^1^	2 ± 2 ab	6 ± 3 a	1 ± 1 a	5 ± 3 a	0.2 ± 0.1 c	0.3 ± 0.1 c	−0.1 ± 0.0 b
	PGase	7 ± 1 a	0 ± 0 a	7 ± 1 a	4 ± 2 ab	1 ± 1 a	3 ± 2 ab	0.3 ± 0.0 bc	0.3 ± 0.1 bc	0.0 ± 0.0 ab
	PLase	0 ± 0 b	0 ± 0 a	0 ± 0 b	0 ± 0 c	1 ± 1 a	−1 ± 1 c	1.1 ± 0.1 a	0.8 ± 0.2 ab	0.4 ± 0.1 a
Hemicellulases	Total	2 ± 1	0 ± 0	2 ± 1	1 ± 1	1 ± 1	0 ± 1	1.7 ± 0.1	1.8 ± 0.2	−0.1 ± 0.1
	XYLase	0 ± 0 b	0 ± 0 a	0 ± 0 b	0 ± 0 bc	0 ± 0 a	0 ± 0 bc	0.8 ± 0.1 ab	0.6 ± 0.0 abc	0.1 ± 0.1 ab
	XGase	1 ± 1 b	0 ± 0 a	1 ± 1 b	1 ± 1 bc	1 ± 1 a	0 ± 1 bc	0.2 ± 0.0 c	0.3 ± 0.1 bc	−0.1 ± 0.0 b
	MANase	2 ± 1 ab	0 ± 0 a	2 ± 1 b	0 ± 9 c	0 ± 0 a	0 ± 0 c	0.8 ± 0.0 ab	0.9 ± 0.1 a	−0.1 ± 0.0 b
Cellulase	CELase	0 ± 0 b	0 ± 0 a	0 ± 0 b	1 ± 1 bc	1 ± 1 a	0 ± 1 bc	0.6 ± 0.1 bc	0.3 ± 0.1 bc	0.3 ± 0.1 ab

^1^Mean separation within columns by Tukey’s Studentized range test, *p* < 0.05.

All enzyme solutions were buffered at their respective pH optima using the manufacturer-recommended buffer system and pH. Buffer only (no enzyme) served as control. The number of cells and protoplasts refers to that contained in 3 µL of the digestion medium. Data represent means ± SE. *N* = 3.

**Figure 6:**
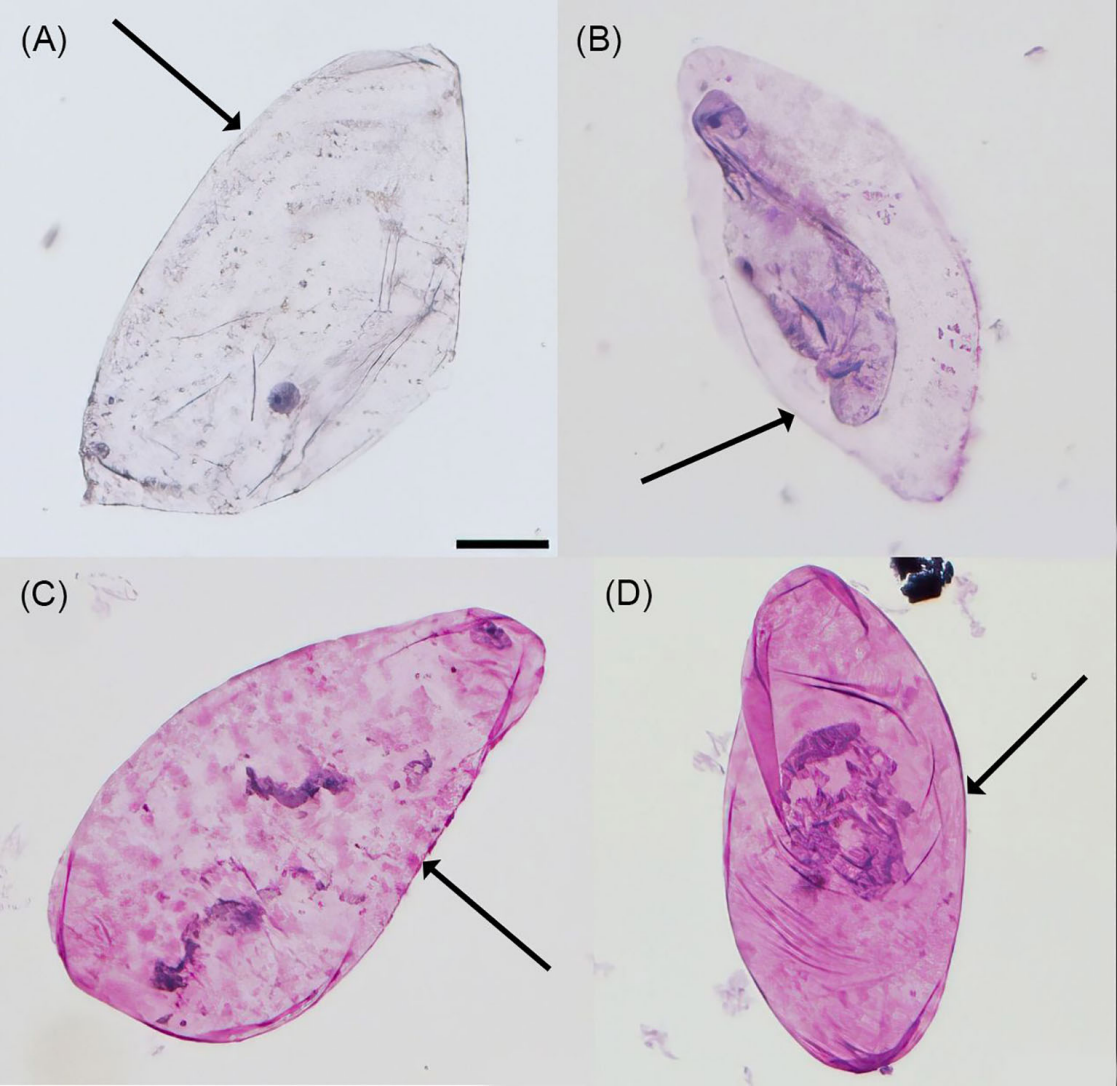
Micrographs showing representative intact cells in the incubation solution when incubating blocks of parenchyma excised from mature “Regina” sweet cherry in buffered solutions of **(A)** endo-1,4-β-D-galactanase (GALase), **(B)** endo-polygalacturonase (PGase), **(C)** endo-1,4-β-xylanase M3 (XYLase), and **(D)** β-mannanase (MANase). All enzyme solutions were buffered at their respective pH optima using the manufacturer-recommended buffer system and pH. Cells were stained with toluidine blue. Edges of cells were sharp when tissue was digested by XYLase and MANase (arrows in C and D), while those of tissue digested with GALase and PGase were soft and blurry (arrows in A and B). Scale bar = 50 µm.

Within the pectinases, PGase was consistently more effective than PLase, while GALase was intermediate. The only exception was the release of protoplasts, where GALase was more effective than PGase. Some cells were also released by the hemicellulases MANase and XGase. The cells released by PGase and GALase had soft cell walls, were fragile, and easily damaged (arrows in **Figures 6A, B**), while those released by MANase and XGase remained intact with sharp edges (arrows in **Figures 6C, D**).

In some cases, the percentage digestion by the enzyme was negative. This was an artifact resulting from the variability of the system when digestion by the buffer and that by the buffer plus enzymes were low, variable, and often not significantly different from zero (**Table 2**).

**Figure 7:**
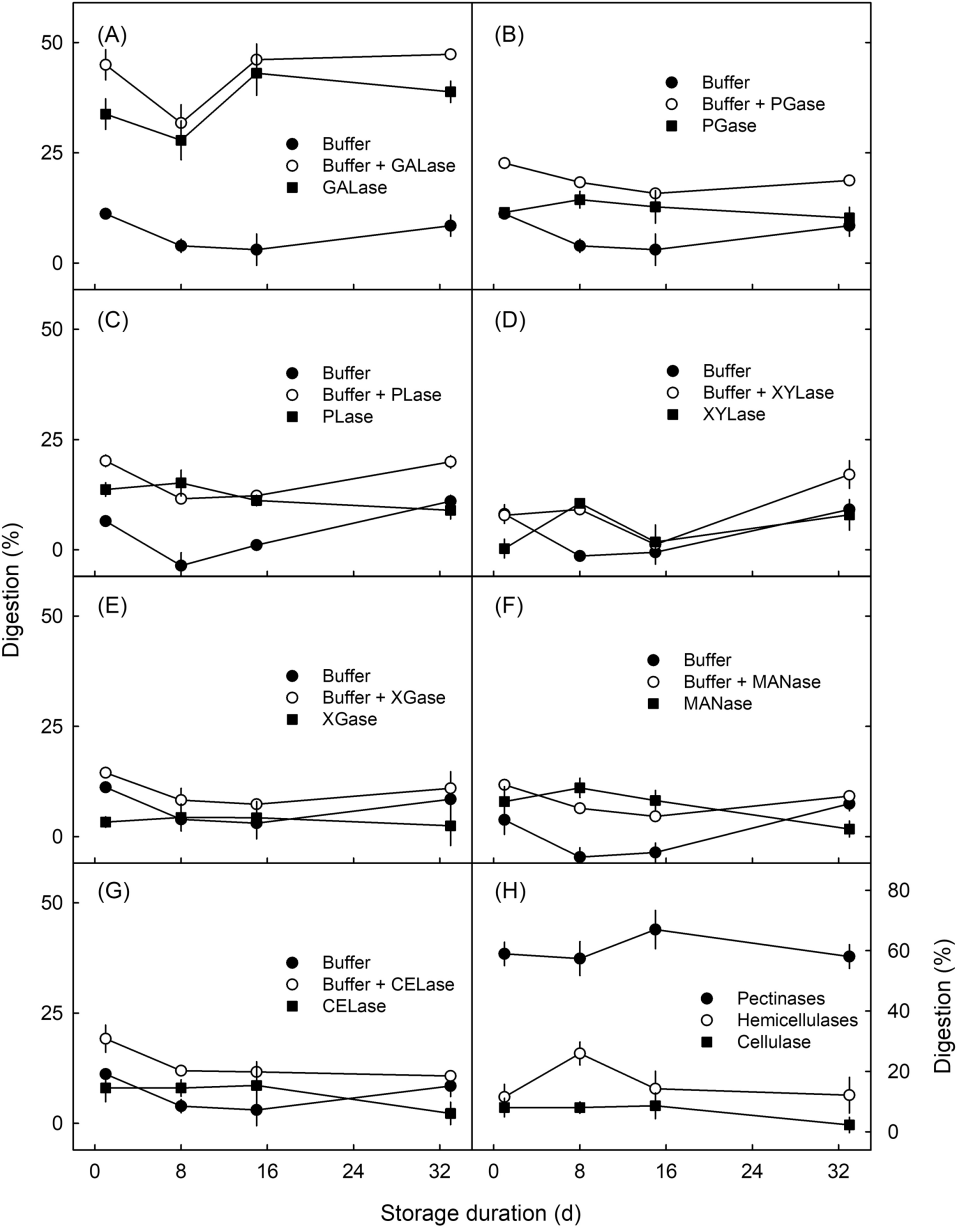
Effect of storage duration on the digestion of parenchyma of mature “Staccato” sweet cherry by various cell wall-degrading enzymes. Enzymes for the pectin fractions were **(A)** endo-1,4-β-D-galactanase (GALase), **(B)** endo-polygalacturonase (PGase), and **(C)** pectate lyase (PLase). Enzymes for the hemicellulose fractions were **(D)** endo-1,4-β-xylanase M3 (XYLase), **(E)** xyloglucanase (XGase), and **(F)** β-mannanase (MANase). For the cellulose fraction, **(G)** endo-cellulase (CELase) was used. **(H)** Cumulative digestion by pectinases, hemicellulases, and cellulase. The fruit were stored at 2°C and 100% relative humidity for between 0 and 33 days. All enzyme solutions were buffered at their respective pH optima using the manufacturer-recommended buffer system and pH. Buffer only (no enzyme) served as control. *N* = 3.

*Effect of storage duration.* There was no consistent effect of storage duration on the digestion of cell walls (**Figures 7A–H**). As in the developmental time course, pectinases were most effective in digesting parenchyma, followed by hemicellulases and cellulase (**Figure 7H**).

*Effect of boiling.* Digestion of parenchyma by native enzymes present in the juice of sweet cherry was of similar order of magnitude to that occurring with the purified enzymes (**Table 3**). Boiling the juice destroyed the cell wall-degrading enzymes as indexed by reduced digestion in boiled juice. With synthetic sweet cherry juice that contained no enzymes, digestion was intermediate (**Table 3**).

**Table 3:** Digestion of parenchyma of mature “Sweetheart” sweet cherry when incubated in its own juice, in its own juice but boiled to inactivate enzymes, or in synthetic juice.

Treatment	Digestion (%)
Juice	28.6 ± 2.0 a^1^
Boiled juice	15.0 ± 1.4 b
Synthetic juice	19.1 ± 0.6 c

^1^Mean separation by Tukey’s Studentized range test, *p* < 0.05.

The synthetic juice comprised glucose, fructose, sorbitol, and lactic acid buffered at pH 3.6 using KOH. These five osmolytes represent the major ones in sweet cherry juice (Herrmann, 2001). Data represent means ± SE. *N* = 3.

*Effect of calcium.* Calcium had a marked effect on the digestibility of parenchyma by the pectinase PGase. Adding Ca significantly reduced digestion by PGase. Extracting and complexing Ca by EGTA significantly increased digestion (**Table 4**). However, pre-incubating intact sweet cherry in CaCl_2_ had no effect on digestion by PGase (**Table 5**). There was no effect of Ca on the digestion by buffer only.

**Table 4:** Effect of CaCl_2_ (10 mM) and EGTA (5 mM) on the digestion of parenchyma of mature “Regina” sweet cherry by endo-polygalacturonase (PGase).

Treatment	Digestion (%)		
	Buffer + PGase	Buffer	PGase
Control	30.4 ± 3.7 ab^1^	19.0 ± 1.7 a	11.4 ± 4.1 a
CaCl_2_	26.2 ± 0.3 a	23.6 ± 0.4 a	2.7 ± 0.5 b
EGTA	37.7 ± 1.3 b	16.7 ± 0.2 a	21.0 ± 1.4 c

^1^Mean separation within columns by Tukey’s Studentized range test, *p* < 0.05.

The PGase enzyme solution was buffered at pH 5.5 using the manufacturer-recommended buffer. Buffer only (no PGase) served as control. Digestion by PGase (“PGase”) was calculated by subtracting the digestion in the buffer (“Buffer”) from that in buffer plus enzyme (“Buffer + PGase”). Data represent means ± SE. *N* = 3.

**Table 5:** Effect of pre-incubating mature “Sweetheart” sweet cherry in 100 mM CaCl_2_ on the digestion of parenchyma by endo-polygalacturonase (PGase).

Pre-incubation	Digestion (%)		
	Buffer + PGase	Buffer	PGase
Control	15.3 ± 1.7 a	−3.4 ± 1.6 a^1^	18.7 ± 2.3 a
CaCl_2_	14.1 ± 1.4 a	−1.7 ± 2.9 a	15.8 ± 3.2 a

^1^Mean separation within columns by Tukey’s Studentized range test, *p* < 0.05.

The enzyme solution was buffered using the manufacturer-recommended buffer and pH. Buffer only (no enzyme) served as control. Digestion by PGase (“PGase”) was calculated by subtracting the digestion in the buffer (“Buffer”) from that in buffer plus enzyme (“Buffer + PGase”). Data represent means ± SE. *N* = 3.

**Figure 8:**
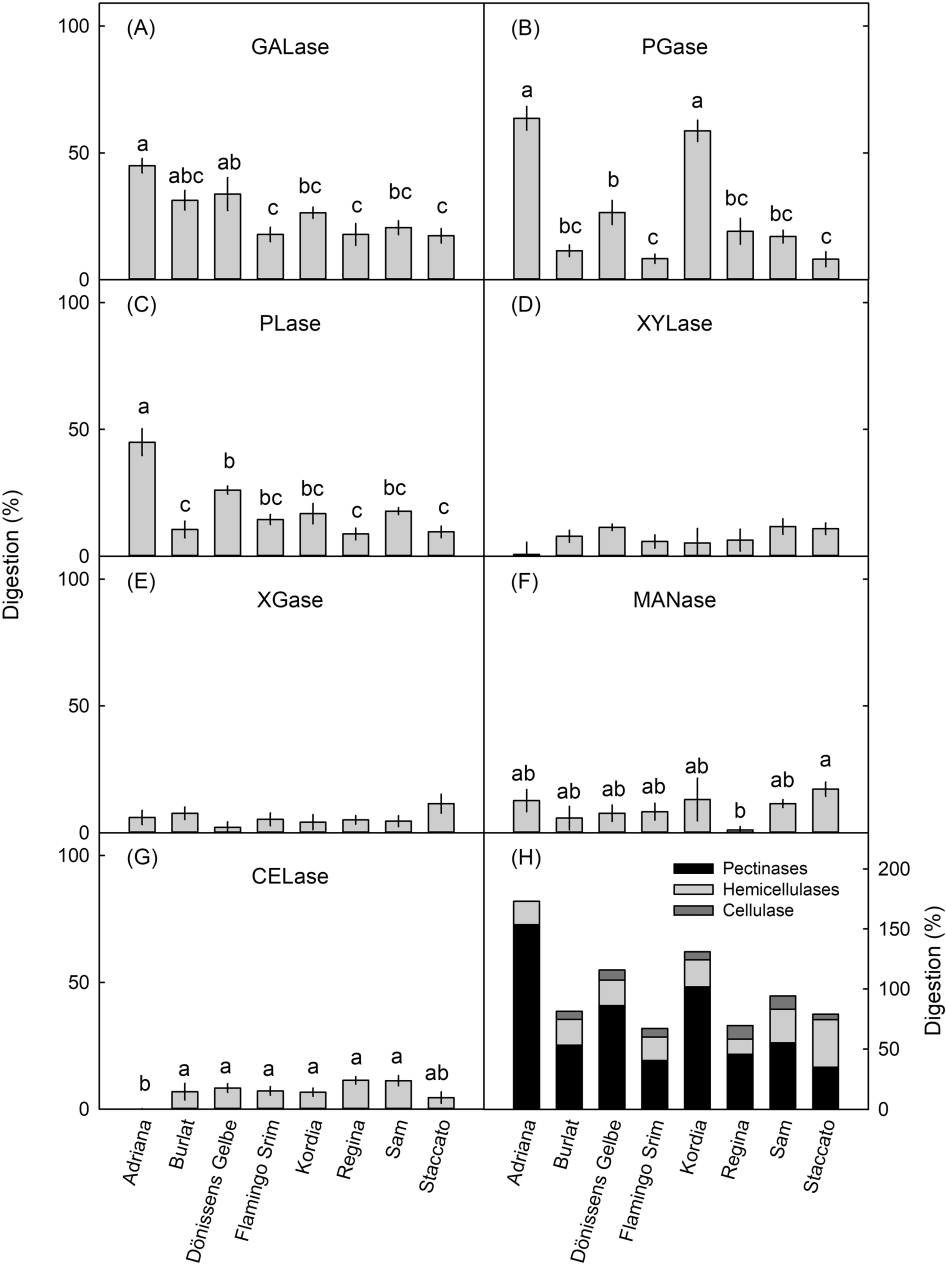
Effect of cultivar on the digestion of parenchyma of sweet cherry cultivars by cell wall-degrading enzymes. The cultivars were “Adriana”, “Burlat”, “Dönissens Gelbe”, “Flamengo Srim”, “Kordia”, “Regina”, and “Staccato”. All fruit were at the fully mature stage. Pectin-degrading enzymes were **(A)** endo-1,4-β-D-galactanase (GALase), **(B)** endo-polygalacturonase (PGase), and **(C)** pectate lyase (PLase). Hemicellulose-degrading enzymes were **(D)** endo-1,4-β-xylanase M3 (XYLase), **(E)** xyloglucanase (XGase), and **(F)** β-mannanase (MANase). **(G)** An endo-cellulase (CELase) was used to degrade cellulose. **(H)** Cumulative digestion by pectinases, hemicellulases, and by cellulase. The fruit were sampled at full maturity. All enzyme solutions were buffered using the manufacturer-recommended buffer system and pH. Buffer only (no enzyme) served as control. Bars labeled with the same letter are not significantly different, Tukey’s studentized range test *p* = 0.05. XYLase and XGase were not significantly different at *p* < 0.05. *N* = 3.

*Effect of cultivar.* The digestion of tissue blocks depended on the cultivar and the specific enzyme (**Figures 8A–H**).

Across cultivars, the pectinases GALase, PGase, and PLase digested more cell walls than the hemicellulases or the cellulase (**Figures 8A–G**). However, there were also clear interactions between cultivars and enzymes, particularly for the pectinases. “Adriana” flesh was digested effectively by GALase, PGase, and PLase, whereas in “Kordia”, PGase was markedly more effective than GALase and PLase. In “Flamengo Srim”, “Regina”, and “Staccato”, digestion by GALase, PGase, and PLase was low. Digestion by the hemicellulases XYLase, XGase, and MANase, as well as by the cellulase CELase, was consistently low and similar across all cultivars (**Figures 8D–G**). It is interesting that across all cultivars, digestion by the three pectinases was significantly and positively cross-correlated, but there were no significant cross-correlations within the hemicellulases or between the pectinases and the hemicellulases (**Table 6**).

**Table 6:** Coefficient of correlation for relationships between the digestion of cell walls by endo-1,4-β-D-galactanase (GALase), endo-polygalacturonase (PGase), pectate lyase (PLase), endo-1,4-β-xylanase M3 (XYLase), xyloglucanase (XGase), β-mannanase (MANase), or endo-cellulase (CELase) in selected sweet cherry cultivars.

Class	Enzyme	Pearson coefficient of correlation (*r*)						
		Pectinases			Hemicellulases			Cellulase
		GALase	PGase	PLase	XYLase	XGase	MANase	CELase
Pectinases								
	PGase	0.687*						
	PLase	0.851***	0.728**					
Hemicelluloses								
	XYLase	−0.483	−0.671*	−0.517				
	XGase	−0.246	−0.348	−0.319	0.054			
	MANase	0.080	0.273	0.260	0.056	0.435		
Cellulase								
	CELase	−0.714**	−0.602	−0.757**	0.670*	−0.298	−0.490	
Cell wall swelling	(µm)	−0.734*	−0.330	−0.685*	0.397	0.507	0.406	0.297

*N* = 8 except for cell wall swelling where *N* = 7. For cell wall swelling data, see **Supplementary Table S1**.

Significance of the coefficient of correlation at the 0.1%, 0.05%, and 0.01% level indicated by *, **, and ***, respectively.

The cultivars were “Adriana”, “Burlat”, “Dönissens Gelbe”, “Flamengo Srim”, “Kordia”, “Regina”, and “Staccato”. All fruit were sampled at full maturity. All enzyme solutions were buffered using the manufacturer-recommended buffer system and pH.

The only exceptions were significant negative correlations between digestion by GALase and CELase or by PLase and CELase and a significant positive correlation between XYLase and CELase.

Correlation analyses further revealed that cell wall swelling was significantly and negatively correlated to GALase and PLase. There were no significant relationships of swelling with hemicellulases or cellulase (**Table 6**).

## Discussion

Our results indicate that (1) pectins are the primary cell wall carbohydrates responsible for cell:cell adhesion in sweet cherry and (2) hemicelluloses are involved in cell:cell adhesion to a lesser extent.

### Pectins are the primary cell wall carbohydrates responsible for cell:cell adhesion

This conclusion is based on the following evidence. First, the pectinases GALase, PGase, and PLase caused the most digestion in the developmental time course experiment (**Figure 4**), in the experiment on the effect of storage duration (**Figure 7**), and (with considerable variability between cultivars) also in the cultivar comparison (**Figure 8**).

Second, digestion by pectinases produced most isolated cells and protoplasts (**Table 2**). Among the pectinases, GALase and PGase were more effective in separating entire cells or isolating protoplasts than PLase. However, PLase produced most cell wall fragments, indicating that pectins are also involved in maintaining the structural integrity of cell walls. PLase digests mostly methylated HG (Brown et al., 2001) whereas PGase digests mostly demethylated HG (Rafique et al., 2021).

Third, we observed significant binding of mAbs to methylated and demethylated HG in skin sections and sections of the parenchyma that had vascular bundles (**Figure 1**). Our findings are also in agreement with the immunolabeling of demethylated HG in cracked sweet cherry fruit published earlier (Schumann et al., 2019). It is not surprising that digestion increased overall as maturity progressed to ripeness and then decreased again as fruit probably became overripe at 72 DAFB. This is likely related to an increasing amount and activity of internal pectinases present in the maturing and ripening fruit that degraded significant amounts of pectin (Barrett and Gonzalez, 1994; Schumann et al., 2019; Zhai et al., 2021).

Fourth, pectins are located in the middle lamella where they are responsible for cell:cell adhesion (Zamil and Geitmann, 2017). Cracking occurs primarily by separation of cells along the middle lamella rather than by rupture of cell walls (Brüggenwirth and Knoche, 2017; Schumann et al., 2019).

Consistent with our observation in sweet cherry is the finding that GALase separates the cells of tomato parenchyma (Ordaz-Ortiz et al., 2009). In tomato, galactans are localized in the primary cell walls and middle lamella (Jones et al., 1997). However, this is not the case in sweet cherry. In sweet cherry, immunolocalization identified galactans only in the vascular tissue (**Figure 1**). There was no binding of mAbs against galactans in the parenchyma. The reason for this is unknown. Potential explanations include a covering/coating of galactan in the cell wall by other cell wall polysaccharides, such as hemicelluloses and cellulose, that may have restricted access of the mAbs against pectins to the parenchyma (Brummell, 2006). This hypothesis would also account for the negative coefficient of correlation between the digestion by hemicellulase (XYLase) and pectinase (PGase) or cellulase (CELase) and pectinases (GALase and PLase).

Together, these considerations support a central role for pectins in cell:cell adhesion in sweet cherry fruit.

### Hemicelluloses are involved to a lesser extent in cell adhesion

Compared to pectins, hemicelluloses play only a minor role in cell adhesion. The hemicellulases were markedly less effective in digesting parenchyma tissue of sweet cherry fruit (**Table 2**). Some cell separation occurred with MANase and XGase, but both were less effective than pectinases. Also, there was essentially no or only weak binding of mAbs for hemicelluloses (**Figure 1**). In this aspect, sweet cherry differs from tomato, where hemicelluloses play a central role in cell:cell adhesion (Ordaz-Ortiz et al., 2009). Among the hemicellulases of tomato, MANase was most effective and even exceeded that of the pectinases GALase, PGase, and PLase (Ordaz-Ortiz et al., 2009). We do not have an explanation for this effect. However, the specificity of the enzyme/epitopes may differ between tomato and sweet cherry. Also, enclosure of hemicelluloses by pectins would limit access to hemicellulases (Marcus et al., 2010). Regardless of the basis for the difference between sweet cherry and tomato, there is no evidence of a major role for hemicelluloses in cell:cell adhesion in sweet cherry.

### Implications for cracking

The question arises whether our findings on cell:cell adhesion are related to rain cracking of sweet cherry in the field. The answer is Yes. First, cracking occurs by separation of cells along the middle lamella rather than by rupture of the cell wall. Loss of cell:cell adhesion—in turn—is caused by cell wall swelling. It is the pectinaceous middle lamellae that swells (Basanta et al., 2013; Schumann et al., 2019). The swelling results from (1) the extraction of Ca from the cell walls by malic acid (Schumann et al., 2022; Winkler et al., 2015), (2) the loss of cell turgor that pressurizes the cell walls and thereby inhibits swelling (Schumann and Knoche, 2020; Schumann et al., 2014), and (3) most likely, the action of internal native cell wall-degrading enzymes that loosen the cell wall as maturation and ripening progress. Our study now reveals that cracking involves failure of pectins. This is consistent with an earlier study by Schumann et al. (2019) who identified pectins on the surfaces of cell walls exposed in microcracks. Also, the increase in digestibility of pectins towards maturity is consistent with increased swelling and increased cracking susceptibility until maturity (Christensen, 1973; Schumann et al., 2022).

Further support for a role of pectins in cracking susceptibility and swelling comes from literature data on the effects of Ca. Calcium is known to lower the susceptibility to cracking (Winkler et al., 2024; Winkler and Knoche, 2019) and to decrease swelling (Schumann et al., 2022). Decreased swelling by Ca improves cell:cell adhesion (Brüggenwirth and Knoche, 2017). Here, we demonstrate that a decrease in swelling by Ca also decreases digestion of cell walls by PGase (**Table 4**). Conversely, increased swelling upon extraction of Ca using EGTA increases digestion by PGase (**Table 4**). According to the eggbox model, Ca crosslinks demethylated HG strains (Grant et al., 1973), which decreases pectin hydration and cell wall swelling (Schumann et al., 2022) and, hence, restricts access of PGase to the pectins. Methylated, demethylated HG, and rhamnogalacturonans are major components of sweet cherry fruit cell walls (Basanta et al., 2014; Schumann et al., 2020). Also, malic acid increases fruit cracking (Winkler et al., 2015). Malic acid extracts Ca from cell walls and, hence, weakens cell:cell adhesion and the strength of cell wall (Winkler et al., 2015).

Our experiments reveal marked differences in digestibility of cell walls between cultivars, particularly for pectinases, whereas digestion by hemicellulases or cellulase was much less variable (**Figure 8**). Furthermore, digestion by pectinases consistently exceeded that by hemicellulases or cellulase. It would be interesting to establish whether these differences among cultivars in digestibility are related to differences in their susceptibilities to cracking. This would be expected, because cell separation precedes the extension of microcracks in the cuticle to macrocracks deep into the tissue. Unfortunately, cracking susceptibility was not investigated in our study. However, data on swelling of cell walls were published earlier for eight of the nine cultivars during two subsequent growing seasons. A preliminary correlation analysis using the limited dataset revealed a significant negative correlation between the digestibility of tissue by GALase or PLase and the increase in swelling of cell walls upon removal of turgor (**Supplementary Figure S2**). A negative correlation implies that low swelling occurs when digestion by GALase and PLase is high and *vice versa*. We do not have a particularly robust explanation for this relationship and cannot exclude an artifact.

First, the relationships between digestibility and swelling were governed by two extremes. “Staccato” is consistently high in swelling of cell walls, but low in digestion by GALase and/or PLase. “Adriana” is consistently low in swelling, but high in digestion by GALase and/or PLase (**Supplementary Figure S2**). Unfortunately, information on cell wall chemistry is not available for these two cultivars. Also, it is not known whether the mechanistic basis of swelling is the same in each cultivar.

Second, the cultivars used for the swelling assays and those used for the digestion assays were from different seasons and sites. Hence, environmental factors differed. These factors could affect the Ca status of the fruit, which, in turn, would affect cell wall swelling and cell wall digestibility.

Third, digestion by natural enzymes already present and active in the maturing fruit may have degraded pectins to a significant extent so that the digestion assay yielded little digestion but high swelling. However, this would be unlikely because swelling is associated with pectins and less so with hemicelluloses and cellulose.

Last, one must always remember that correlation does not prove causation.

The above arguments indicate that, based on the limited dataset available, a robust conclusion regarding a mechanistic relationship between cell wall digestibility and cell wall swelling cannot, at present, be drawn. To more certainly identify such a relationship, a matching dataset on digestion, on cell wall swelling and cell wall chemistry, and on cell wall-bound Ca should be generated on a larger number of sweet cherry cultivars. Only then can the mechanistic relationships be more robustly established. Nevertheless, for now, the above is, at worst, a plausible hypothesis.

## Conclusions

Our results demonstrate that pectins are primarily involved in cell:cell adhesion in sweet cherry fruit, whereas hemicelluloses play only a minor role. This finding underlines the critical role of pectins in the separation of neighboring cells in the cracking process. Given the economic importance of cracking of sweet cherry and other fruit crops worldwide, this topic merits further study. In particular, relationships between cell wall chemistry, Ca status, and cell:cell adhesion should be explored.

## Data Availability

The raw data supporting the conclusions of this article will be made available by the authors, without undue reservation.
