# Supplementary material for: Cell:cell adhesion in sweet cherry fruit primarily due to pectins
Source: Front Plant Sci. 2026 Jan 30;16:1690728. doi: 10.3389/fpls.2025.1690728 (PMC12901442; doi:10.3389/fpls.2025.1690728)
Supplement: Supplementary file 1 [file Table1.docx]

# Supplementary Tables

| **Table S1.** Mass, osmolarity, dry matter content, color, and cell wall swelling of ‘Adriana’, ‘Burlat’, ‘Dönissens Gelbe’, ‘Flamengo Srim’, ‘Kordia’, ‘Regina’, ‘Sam’, and ‘Staccato’ sweet cherry. | | | | | | | |
| --- | --- | --- | --- | --- | --- | --- | --- |
| Cultivar | Mass | Osmolarity | Dry matter content | Color | Cell wall swelling^1^ (µm) | | |
|  | (g) | (mmol kg^-1^) | (mg g^-1^ FM) | (°Hue) | 2015 | 2016 | Mean |
| Adriana | 9.8 ± 0.4 | 899 ± 25 | 95 ± 1 | 15 ± 1 | 1.8 ± 0.2 | 2.1 ± 0.2 | 2.1 ± 0.2 |
| Burlat | 9.1 ± 0.4 | 1155 ± 49 | 125 ± 2 | 16 ± 0 | 2.5 ± 0.2 | 2.5 ± 0.2 | 2.5 ± 0.2 |
| Dönissens Gelbe | 6.6 ± 0.2 | 1351 ± 48 | 132 ± 5 | 85 ± 1 | 2.6 ± 0.2 | 2.6 ± 0.2 | 2.6 ± 0.2 |
| Flamengo Srim | 7.8 ± 0.2 | 1370 ± 40 | 154 ± 3 | 50 ± 3 | n.d. | n.d. | n.d. |
| Kordia | 11.3 ± 0.6 | 1081 ± 56 | 115 ± 3 | 14 ± 0 | 4.0 ± 0.2 | 4.0 ± 0.2 | 4.0 ± 0.2 |
| Regina | 9.2 ± 0.5 | 1356 ± 56 | 143 ± 3 | 13 ± 1 | 3.1 ± 0.3 | 3.1 ± 0.3 | 3.1 ± 0.3 |
| Sam | 8.0 ± 0.3 | 1158 ± 44 | 134 ± 6 | 15 ± 0 | 3.0 ± 0.2 | 3.0 ± 0.2 | 3.0 ± 0.2 |
| Staccato | 10.3 ± 0.4 | 1280 ± 50 | 150 ± 5 | 18 ± 0 | 4.7 ± 0.3 | 4.7 ± 0.3 | 4.7 ± 0.3 |
| Data represent means ± SE, N = 10, n.d.= not determined  ^1^ Data for cell wall swelling taken from Schumann et al. (2020) | | | | |  |  |  |
