# Supplementary material for: Cell:cell adhesion in sweet cherry fruit primarily due to pectins
Source: Front Plant Sci. 2026 Jan 30;16:1690728. doi: 10.3389/fpls.2025.1690728 (PMC12901442; doi:10.3389/fpls.2025.1690728)
Supplement: Supplementary file 2 [file SupplementaryFile1.docx]

Supplementary Material

# Supplementary Figures


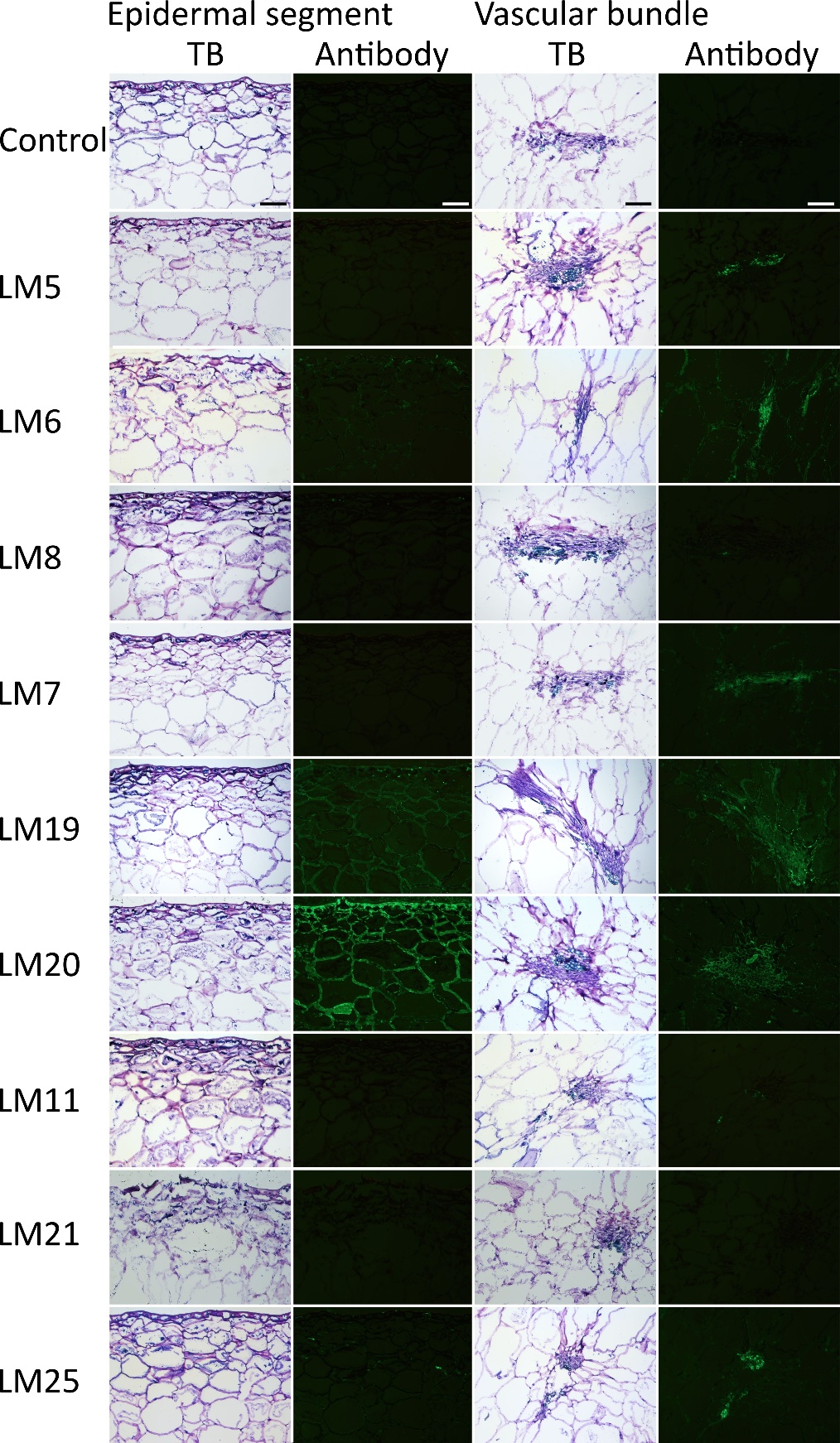


**Supplementary Figure 1.** Immunolocalisation of cell wall epitopes in the skin and parenchyma of cross-sections of the cherry cultivar Adriana. Monoclonal antibodies (mAbs) were for the pectin fractions galactan (LM5), arabinan (LM6), xylogalacturonan (LM8), non-blockwise methylated homogalacturonan (HG) (LM7), demethylated HG (LM19), blockwise methylated HG (LM20) and for the hemicellulose fractions xylan/arabinoxylan (LM11), mannan (LM21) and xyloglucan (LM25). Light micrographs taken in brightfield stained with toluidine blue (TB). Scale bar = 100 µm.


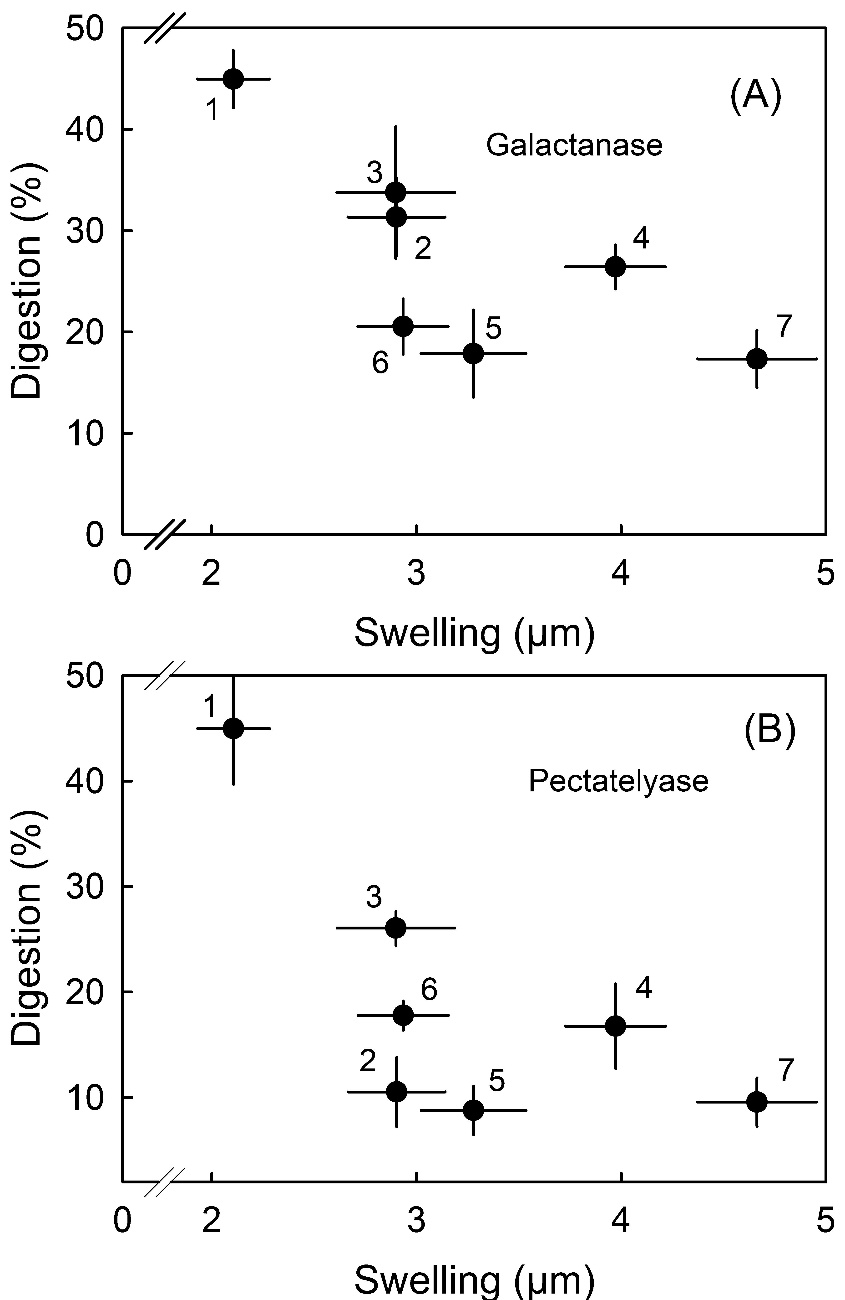


**Supplementary Figure 2.** Relationship between the digestion of parenchyma tissue of selected cultivars by **(A)** galactanase or **(B)** pectate lyase and cell wall swelling. Data for cell wall swelling is from Schumann and Knoche (2020). Swelling data represent the mean swelling of cell walls in two consecutive growing seasons (Schumann and Knoche, 2020). The cultivars were Adriana **(1)**, Burlat **(2)**, Dönissens Gelbe **(3)**, Kordia **(4)**, Regina **(5)**, Sam **(6)** and Staccato **(7)**.
